# PATZ1 knockdown enhances malignant phenotype in thyroid epithelial follicular cells and thyroid cancer cells

**DOI:** 10.18632/oncotarget.19787

**Published:** 2017-08-02

**Authors:** Asumi Iesato, Teruo Nakamura, Hiroto Izumi, Takeshi Uehara, Ken-Ichi Ito

**Affiliations:** ^1^ Division of Breast, Endocrine and Respiratory Surgery, Department of Surgery (II), Shinshu University School of Medicine, Matsumoto, Japan; ^2^ Department of Occupational Pneumology, Institute of Industrial Ecological Sciences, University of Occupational and Environmental Health, Kitakyushu, Japan; ^3^ Department of Laboratory Medicine, Shinshu University School of Medicine, Matsumoto, Japan

**Keywords:** thyroid cancer, PATZ1, dedifferentiation, invasion, migration

## Abstract

This study was designed to examine the involvement of PATZ1 in carcinogenesis and dedifferentiation of thyroid cancer. Immunohistochemistry on clinical specimens indicated nuclear PATZ1 expression in all normal thyroid glands and adenomatous goiter, while nuclear PATZ1 expression decreased along with the dedifferentiation of thyroid cancer. Knockdown of nuclear PATZ1 by siRNA in an immortalized normal follicular epithelial cell line (Nthy-ori 3-1) altered cellular morphology and significantly increased cell proliferation, migration, and invasion. In addition, the expression of urokinase-type plasminogen activator (uPA), matrix metalloproteinase (MMP) 2, MMP9, and MMP11 was increased by PATZ1 knockdown in Nthy-ori 3-1 cells. When PATZ1 was silenced in differentiated thyroid cancer (DTC) cell lines (TPC-1 and FTC-133), proliferation, cellular motility, and expression of uPA and MMPs were significantly increased. Forced expression of exogenous PATZ1 decreased proliferation, cellular motility, and the expression of uPA and MMPs in ATC cell lines (ACT-1 and FRO). In thyroid cancer cell lines, PATZ1 functioned as a tumor suppressor regardless of p53 status. Moreover, the ratio of nuclear PATZ1 positive tumors was significantly decreased in ATC irrespective of p53 status. Our study demonstrates that PATZ1 knockdown enhances malignant phenotype both in thyroid follicular epithelial cells and thyroid cancer cells, suggesting that PATZ1 functions as a tumor suppressor in thyroid follicular epithelial cells and is involved in the dedifferentiation of thyroid cancer.

## INTRODUCTION

Thyroid cancer is the most common endocrine malignancy worldwide [1]. More than 95% of thyroid cancers are derived from thyroid follicular epithelial cells and are classified into differentiated thyroid cancer (DTC), including papillary and follicular thyroid cancer (PTC and FTC), poorly differentiated thyroid cancer (PDTC), and anaplastic thyroid cancer (ATC). DTC behaves in an indolent manner and usually shows a favorable prognosis. In contrast, ATC is one of the most aggressive cancers in humans with a median survival rate of 3–9 months and is responsible for 14–39% of thyroid cancer-related deaths regardless of the multimodal treatments, although it accounts for less than 2% of all thyroid cancers [2, 3].

The clinical course of DTC and pre- or co-existence of DTC and less differentiated cancers such as PDTC or ATC in clinical specimens indicate the sequential progression of DTC to more aggressive cancers [4–6]. On the other hand, recent advances revealed a relatively high prevalence of genetic alterations in the *RET-Ras-BRAF* signaling cascade and other unique chromosomal rearrangements in thyroid cancer and demonstrated that most PDTC or ATC derive from pre-existing well-differentiated thyroid cancer through additional genetic alterations, including β-catenin nuclear accumulation and p53 inactivation [7]. However, the underlying molecular mechanisms of the sequential progression of DTC to more aggressive phenotypes such as PDTC or ATC remain poorly understood. Therefore, elucidation of the mechanisms underlying the progression from indolent DTC to more aggressive PDTC and ATC may lead to the development of novel therapeutic strategies for the aggressive phenotype of thyroid cancers, consequently reducing the number of death due to thyroid cancer.

In an effort to elucidate the underlying molecular mechanisms of the transition from indolent DTC to virulent ATC, we reported the altered expression of several molecules such as UDP-GalNAc: polypeptide N-acetylgalactosaminyl transferases-3 (GalNAc-T3) and epithelial cell adhesion molecule (EpCAM) together with CD44v6 and claudin-7 as well as aldehyde dehydrogenase 1 (ALDH1) in the development of the aggressive phenotype of thyroid cancer [6, 8]. In order to detect molecules whose expression changes during the transition to a more aggressive phenotype, we compared gene expression profiles by microarray analysis between DTC and ATC components in clinical specimens obtained from the same patients and demonstrated the drastic alteration of POZ/BTB and AT-hook-containing zinc finger protein 1 (PATZ1) expression during anaplastic transformation.

PATZ1, also named zinc finger protein 278 (ZNF278), MAZ-related factor (MAZR), or zinc finger sarcoma gene (ZSG), is an ubiquitously expressed transcriptional regulatory factor gene whose product binds to the RING finger protein 4 (RNF4) that associates with a variety of transcription regulators [9, 10]. PATZ1 is a member of the POZ and Kruppel-like zinc finger (POK) family and is able to either activate or repress gene transcription depending on the cellular context [9, 11–13]. Although the physiological role of PATZ1 has not been fully elucidated, recent studies demonstrated that PATZ1 plays critical roles in spermatogenesis [14], embryonic development [13], apoptosis [13, 15], cell proliferation [13, 16, 17], cell senescence [13, 18], and DNA damage response [17].

With regard to cancer, several studies indicated the involvement of PATZ1 in carcinogenesis. However, both oncogenic and tumor suppressor roles have been reported. PATZ1 overexpression has been described in various human malignant neoplasms, including colon, testicular, and breast tumors, suggesting an oncogenic role of PATZ1 [14, 16, 19]. On the other hand, other studies suggested that PATZ1 acts as a tumor suppressor by interacting with p53 and regulating the function of p53-target genes [13, 18]. Regarding thyroid cancer, Chiappetta *et al.* recently reported that PATZ1 was downregulated in a large panel of thyroid cancer samples and cell lines, and that restoration of PATZ1 in thyroid cancer cell lines decreased migration, epithelial-mesenchymal transition, and *in vivo* tumorigenic potential, which demonstrates a tumor suppressor role of PATZ1 in the development of thyroid cancer [20]. However, the mechanisms underlying the role of PATZ1 in carcinogenesis of thyroid epithelial cells and progression of thyroid cancer remain unclear.

The purpose of this study was to investigate the role of PATZ1 in carcinogenesis of thyroid follicular epithelial cells and the mechanisms underlying the progression of thyroid cancer to more aggressive phenotype. We demonstrated that PATZ1 is involved in the transition of normal thyroid follicular epithelial cells to malignant phenotype as well as in the dedifferentiation of thyroid cancer cells by altering the expression of proteolytic enzymes. Our study suggests that PATZ1 might be involved in the oncogenic process of thyroid cancer from its early to late stage.

## RESULTS

### PATZ1 expression in clinical specimens

**Figure 1:**
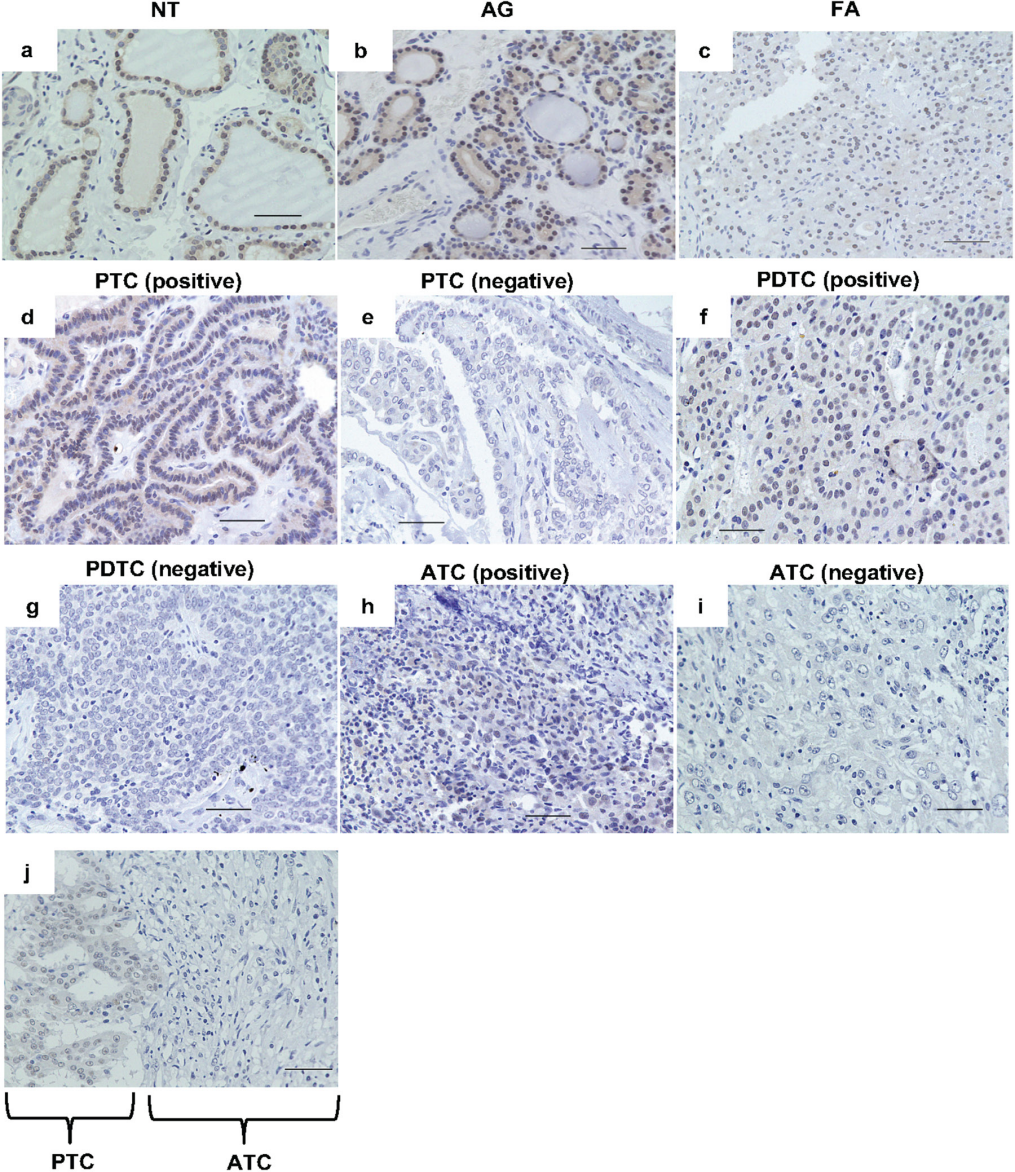
PATZ1 expression assessed by immunohistochemistry (A) PATZ1 expression was evaluated by immunohistochemistry in normal thyroid gland (NT) **(a)**, adenomatous goiter (AG) **(b)**, follicular adenoma (FA) **(c)**, papillary thyroid cancer (PTC) **(d)** positive, (**e**; negative), poorly differentiated thyroid cancer (PDTC) (**f**; positive, **g**; negative), and anaplastic thyroid cancer (ATC) (**h**; positive, **i**; negative), and transitional lesion from PTC to ATC (**j**). Scale bar = 50 μm.

We analyzed nuclear PATZ1 expression by immunohistochemistry in 160 thyroid clinical tissues, including 50 normal thyroid tissues (NT), 18 adenomatous goiters (AG), 5 follicular adenomas (FA), 39 PTC, 8 FTC, 12 PDTC, and 28 ATC. As shown in Table 1, all NT and AG were positive for nuclear PATZ1. In PTC, positive nuclear staining for PATZ1 was observed in 35 of 39 tumors (89.7%), whereas in FTC, the positive staining for PATZ1 was detected in 5 of 8 tumors (62.5%). Positive nuclear staining for PATZ1 in PDTC was observed in 7 of 12 tumors (58.3%). In contrast, only 3 of 28 (10.7%) ATC specimens were positive for nuclear PATZ1. The frequency of positive nuclear PATZ1 expression decreased as the dedifferentiation of thyroid cancer progressed, and the frequency of nuclear PATZ1 expression in ATC was significantly decreased compared to that in NT/AG, DTC (i.e., PTC and FTC), or PDTC. These results suggest that PATZ1 may be involved in both carcinogenesis and progression of thyroid cancer. Representative findings of NT, AG, FA, and both of positive and negative DTC, PDTC, and ATC cases are presented in Figure 1.

**Table 1:** Expression of nuclear PATZ1 in thyroid tumors

Histological type		NT (N = 50)	AG (N = 18)	FA (N = 5)	PTC (N = 39)	FTC (N = 8)	PDTC (N = 12)	ATC (N = 28)
**PATZ1**	positive (%)	50 (100)	18 (100)	4 (80.0)	35 (89.7)	5 (62.5)	7 (58.3)	3^*#$^ (10.7)
	negative (%)	0 (0)	0 (0)	1 (20.0)	4 (37.5)	3 (37.5)	5 (41.7)	25 (89.3)

NT; normal thyroid gland, AG; adenomatous goiter, FA; follicular adenoma, PTC; papillary thyroid cancer, FTC; follicular thyroid cancer, PDTC; poorly differentiated thyroid cancer, ATC; anaplastic thyroid cancer.

*p < 0.01 *vs*. NT/AG, ^#^*p* < 0.01 *vs*. DTC (PTC/FTC), ^$^*p* < 0.01 *vs*. PDTC.

### Expression of PATZ1, uPA, MMPs, and p53 in an immortalized normal thyroid epithelial cell line and thyroid cancer cell lines

**Figure 2:**
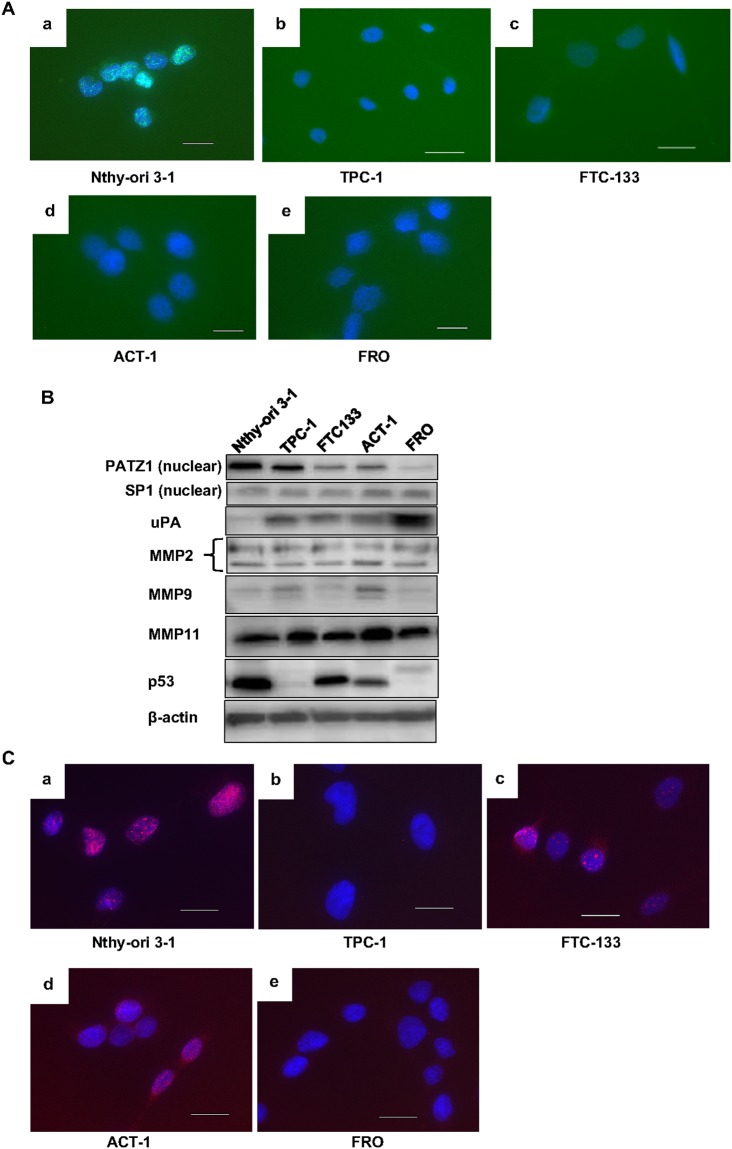
Characteristics of the cell lines **(A)** Immunofluorescence analysis of PATZ1 expression in cell lines. The cells were labeled by using PATZ1 antibody (green) and examined under a fluorescence microscope. Nuclei were stained with DAPI (blue). A representative image for each cell line is shown; Nthy-ori 3-1 **(a)**, TPC-1 **(b)**, FTC-133 **(c)**, ACT-1 **(d)**, and FRO **(e)**. Scale bar = 25 μm. **(B)** Expression of nuclear PATZ1, uPA, MMPs, and p53 in five cell lines. Nuclear extracts (PATZ1 and SP1) and whole cell lysates (uPA, MMPs, and p53) were analyzed by western blot analysis. SP1 and β-actin were used as internal loading controls. **(C)** Immunofluorescence analysis of p53 expression in the cell lines. The cells were labeled by using p53 antibody (red). Nuclei were stained with DAPI (blue). A representative image of each cell line is shown; Nthy-ori 3-1 **(a)**, TPC-1 **(b)**, FTC-133 **(c)**, ACT-1 **(d)**, and FRO **(e)**. Scale bar = 25 μm.

First, to confirm that the cell lines used in the study maintained the same characteristics regarding PATZ1 as clinical thyroid specimens, we examined PATZ1 cellular localization in an immortalized normal thyroid epithelial cell line, Nthy-ori 3-1, and four thyroid cancer cell lines (TPC-1, FTC-133, FRO, and ACT-1) by immunofluorescence (Figure 2A). In Nthy-ori 3-1 cells, PATZ1 was strongly expressed in the nucleus, while a weak expression was detected in the cytoplasm. In TPC-1 and FTC-133 cells, PATZ1 expression was detected in both the nucleus and cytoplasm. However, PATZ1 expression in the nucleus was obviously weaker than that in Nthy-ori 3-1 cells. Only a faint expression was observed in both the nucleus and cytoplasm in the ATC cell lines, FRO and ACT-1. Western blot analysis indicated that the nuclear PATZ1 expression in Nthy-ori 3-1 cells was higher than that in thyroid cancer cell lines, and the nuclear PATZ1 expression in TPC-1 that originated from DTC was higher than that in the ATC cell lines (ACT-1 and FRO) (Figure 2B). These results indicate that the cell lines used in this study maintained the same characteristics regarding PATZ1 expression as clinical specimens and suggest that loss of nuclear localization of PATZ1 may be associated with carcinogenesis and dedifferentiation of thyroid cancer.

Next, the expression of uPA, MMP2, MMP9, and MMP11 at a steady state was examined by western blot analysis in these cell lines (Figure 2B). uPA expression was higher in cancer cell lines compared to that in the immortalized normal thyroid epithelial cell line, Nthy-ori 3-1 cells. uPA expression in FRO was stronger than that in the other cell lines. With regard to MMP2, all cancer cell lines except ACT-1 cells showed equivalent expression levels to that in Nthy-ori 3-1 cells. MMP9 and MMP11 expression levels in FTC-133 and FRO cells were equivalent to that in Nthy-ori 3-1, while MMP9 and MMP11 expression was higher in TPC-1 and ACT-1 than in Nthy-ori 3-1 cells. uPA expression was the highest in FRO cells, and that of MMP 2, 9, and 11 was the highest in ACT-1 cells.

As PATZ1 interacts with p53, we examined p53 expression in the cell lines by western blot and immunofluorescence analyses. Sequence analyses confirmed that Nthy-ori 3-1, TPC-1, and ACT-1 cells harbor wild-type p53, whereas FTC-133 cells harbor mutated p53 (CGT->CAT R273H) and FRO cells do not express p53 [21–24]. In the present study, p53 expression was detected in Nthy-ori 3-1, FTC-133, and ACT-1 cells both by western blot and immunofluorescence analyses, while the expression of p53 was not detected in TPC-1 and FRO cells (Figure 2B, 2C). These results were consistent with results from the previous studies. A relatively higher p53 expression was observed in Nthy-ori 3-1 cells. This may be associated with the fact that the cells were immortalized by transfection of SV40 large T gene that stabilizes p53 [25, 26].

### PATZ1 knockdown induces morphological changes in the immortalized normal thyroid epithelial cell line

**Figure 3:**
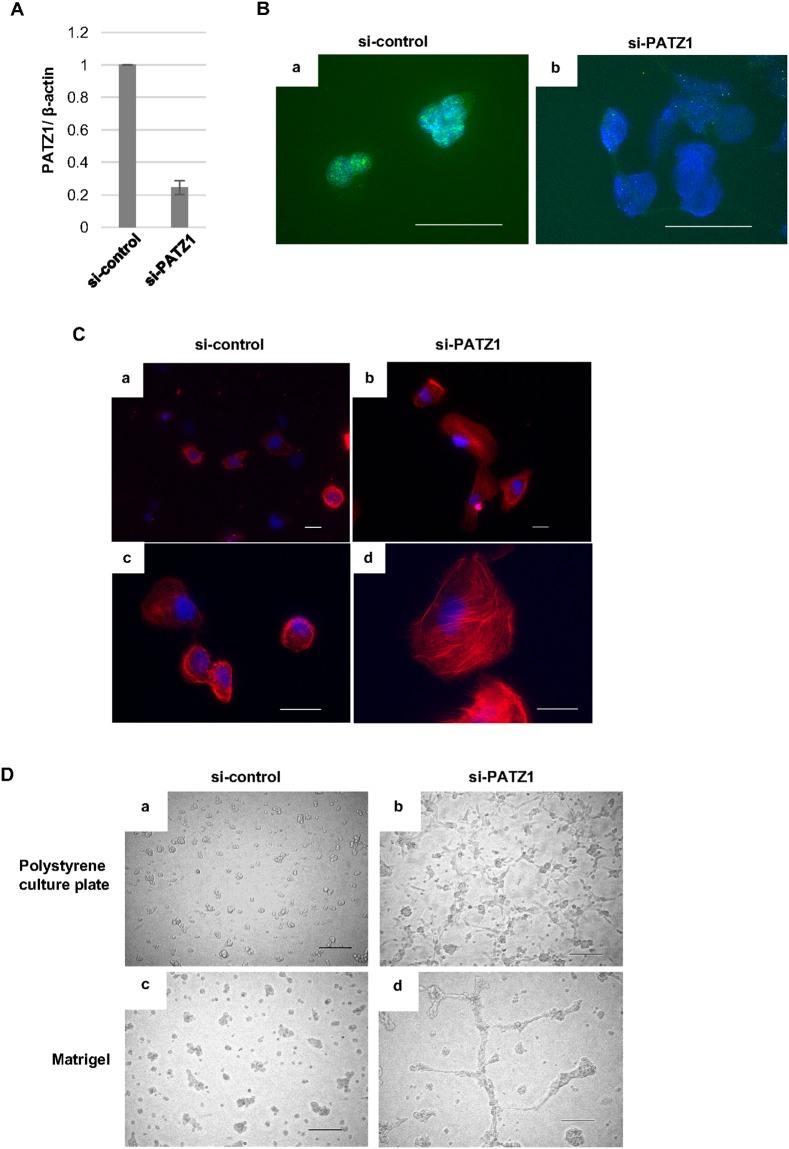
Effects of PATZ1 knockdown on the morphology of immortalized human thyroid epithelial cells, Nthy-ori 3-1 cells **(A)** Relative *PATZ1* mRNA expression in Nthy-ori 3-1 cells transfected with control siRNA (si-control) or siRNA targeting PATZ1 (si-PATZ1). Expression levels were normalized to that of β-actin. **(B)** Immunofluorescence analysis of PATZ1 expression in Nthy-ori 3-1 cells transfected with si-control **(a)** or si-PATZ1 **(b)**. PATZ1 was stained in green. Nuclei were stained with DAPI (blue). Scale bar = 50 μm. **(C)** Immunofluorescence analysis of F-actin in Nthy-ori 3-1 cells transfected with si-control **(a, c)** or si-PATZ1 **(b, d)**. Representative pictures at 400× magnification (a, b) and 1000× magnification are shown. F-actin was visualized by staining with rhodamine-phalloidin (red). Nuclei were stained with DAPI (blue). Scale bar = 25μm. **(D)** Morphological change of Nthy-ori 3-1 cells transfected with si-control or si-PATZ1. Representative images of the cells on polystyrene tissue culture plate **(a, c)** and Matrigel coated plate **(b, d)** 24 h after seeding are shown. Scale bar = 200 μm.

In order to examine the function of PATZ1 in normal thyroid follicular epithelial cells, we silenced PATZ1 expression by siRNA in the immortalized normal thyroid follicular epithelial cell line, Nthy-ori 3-1 cells and then tested whether or not PATZ1 depletion would alter the morphology of the cells. PATZ1 depletion by siRNA was confirmed at the mRNA levels by RT-PCR (Figure 3A). In addition, the decrease of nuclear PATZ1 localization was confirmed by immunofluorescence analysis (Figure 3B). In the F-actin staining with rhodamine-conjugated phalloidin, faint F-actin stress fibers stretched multidirectionally in the Nthy-ori 3-1 cells transfected with control siRNA. In contrast, the F-actin stress fibers were aligned in lamellipodia-like and dot-like structures in the cells transfected with PATZ1 siRNA. Interestingly, the size of the cells increased when nuclear PATZ1 was depleted by siRNA (Figure 3C).

With regard to the morphology of the cells, we tested the effect of PATZ1 inhibition using ordinary polystyrene culture plate and Matrigel (Figure 3D). When Nthy-ori 3-1 cells transfected with PATZ1 siRNA were seeded on the polystyrene tissue culture plate, morphological changes with filopodia-like dendritic protrusions were observed. In contrast, the filopodia-like dendritic protrusions became more prominent and crosslinks between the cells with dendritic protrusions were formed when PATZ1 knocked down Nthy-ori 3-1 cells were seeded on Matrigel.

Thus, PATZ1 depletion induced morphological changes with cytoskeletal alteration in the immortalized normal thyroid epithelial cell line.

### PATZ1 knockdown increases cell proliferation, migration, and invasion in parallel with upregulation of uPA and MMPs in the immortalized normal thyroid epithelial cell line

In order to examine whether PATZ1 was involved in the proliferation and motility of thyroid follicular epithelial cells, we silenced PATZ1 expression by siRNA in Nthy-ori 3-1 cells and assessed cell proliferation, migration, and invasion.

**Figure 4:**
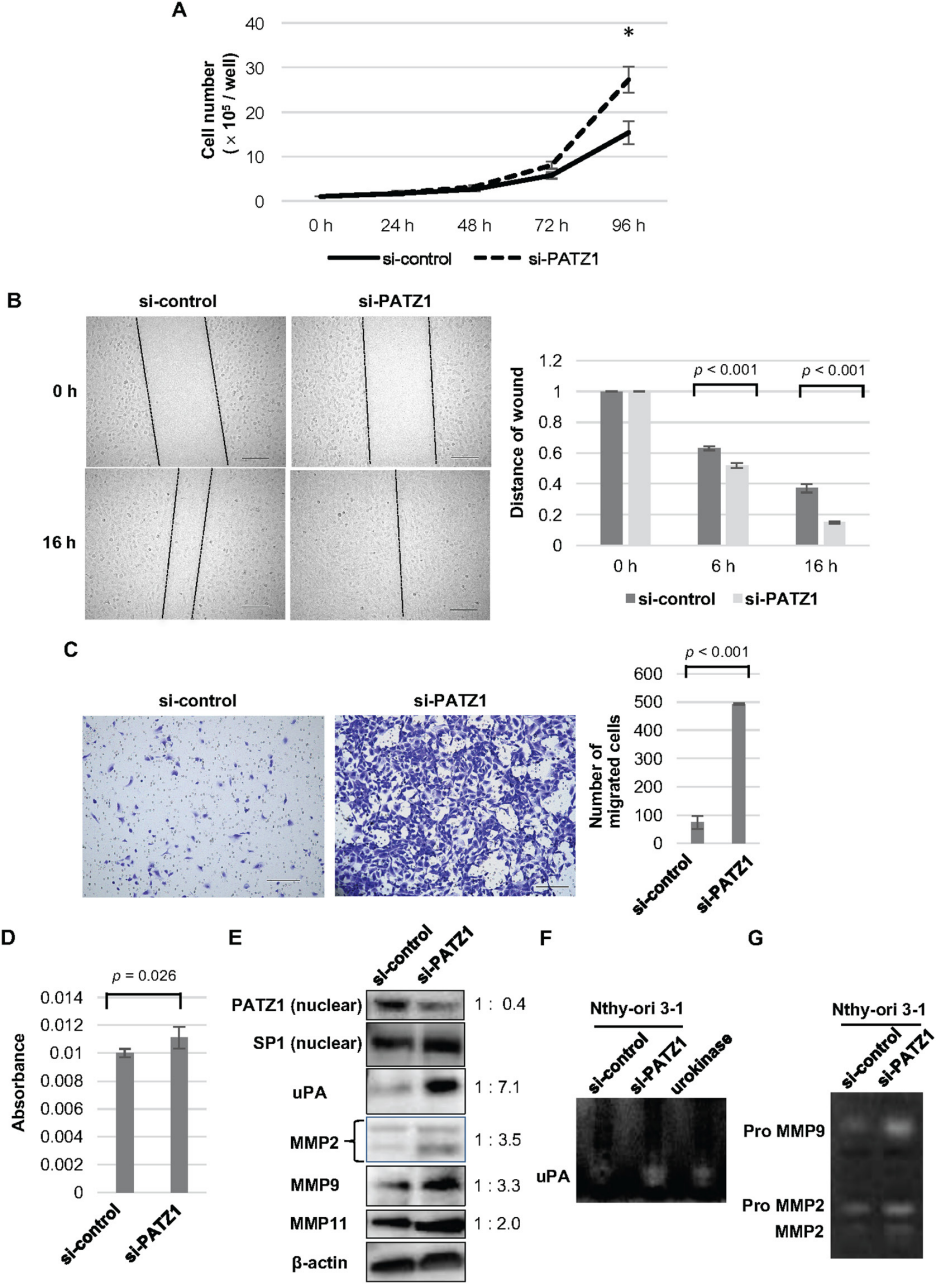
Effects of PATZ1 knockdown on cell proliferation, migration, and invasion in Nthy-ori 3-1 cells **(A)** Cell proliferation assay for Nthy-ori 3-1 cells transfected with control siRNA (si-control) or siRNA targeting PATZ1 (si-PATZ1). The number of cells was counted every 24 h until 96 h after seeding the cells. The number of cells is shown in the bar chart. *p < 0.05. **(B)** Scratch wound assay for Nthy-ori 3-1 cells transfected with si-control or si-PATZ1. Representative images of scratch wound assay in Nthy-ori 3-1 cells transfected with si-control or si-PATZ1 at 0 h and 16 h after a confluent cell monolayer was scratched (left panel, scale bar = 200 μm). The length of the gap at 100 points was measured for each sample and the average ratio of residual gap to the initial gap is shown in the bar chart (right panel). **(C)** Chamber migration assay for Nthy-ori 3-1 cells transfected with si-control or si-PATZ1. Representative images of migrating cells stained with crystal violet in Nthy-ori 3-1 cells transfected with si-control or si-PATZ1 at 24 h (left panel, scale bar = 200 μm). The number of migrated cells is shown in the bar chart (right panel). **(D)** Chamber-invasion assay for Nthy-ori 3-1 cells transfected with si-control or si-PATZ1. The cells that invaded through the transwell chamber coated with collagen type IV were counted as described in Materials and Methods. The absorbance values are shown in the bar chart. **(E)** The expression of PATZ1, uPA, and MMPs in Nthy-ori 3-1 cells transfected with si-control or si-PATZ1. A representative western blot is shown. SP1 and β-actin were used as internal loading control for nuclear extract and whole cell lysate, respectively. **(F)** Activity of uPA in Nthy-ori 3-1 cells transfected with si-control or si-PATZ1. A representative picture of fibrin zymography is shown. Urokinase was used as a positive control and the enzymatic activity was detected at 42 kDa. **(G)** Activities of MMP2 and MMP9 in Nthy-ori 3-1 cells transfected with si-control or si-PATZ1. A representative picture of gelatin zymography is shown. Degradation of gelatin is detected at approximately 92 kDa (Pro MMP9), 72 kDa (ProMMP2), and 66 kDa (MMP2).

Proliferation of Nthy-ori 3-1 cells was significantly increased by PATZ1 knockdown after 72 hours of incubation. (Figure 4A).

Cell migration was tested by scratch assay (Figure 4B) and Boyden chamber migration assay (Figure 4C). In the scratch assay, PATZ1 knockdown significantly increased Nthy-ori 3-1 cell migration compared with that of the cells transfected with control siRNA. In Boyden chamber migration assay, the number of cells that migrated through the pores increased more than six-folds in the Nthy-ori 3-1 cells transfected with PATZ1 siRNA compared to that in the Nthy-ori 3-1 cells transfected with control siRNA.

We examined cell invasion ability using Boyden chamber invasion assay (Figure 4D). In the invasion assay, type IV collagen was used as the extracellular matrix (ECM) coated onto the membrane. A significant increase of invasion ability was observed in Nthy-ori 3-1 cells knocked down for PATZ1. These results suggest that PATZ1 knockdown increases both proliferation and motility of thyroid follicular epithelial cells.

As PATZ1 knockdown increased the motility of PATZ1 cells, the expression of proteins related to cell migration and invasion was evaluated by western blot analysis (Figure 4E). PATZ1 knockdown upregulated the expression of uPA, MMP2, MMP9, and MMP11 in Nthy-ori 3-1 cells. In addition, the enzymatic activity of uPA was evaluated by fibrin zymography (Figure 4F), while that of MMP2 and MMP9 was by gelatin zymography (Figure 4G). Zymography showed that uPA, MMP2, and MMP9 activities were increased by PATZ1 knockdown. These results suggest that loss of PATZ1 function increases cellular motility of thyroid follicular epithelial cells through upregulation of uPA and MMPs, leading to the degradation of the ECM.

### Effects of PATZ1 knockdown in differentiated thyroid cancer cell lines

Immunohistochemistry on clinical specimens revealed that PATZ1 nuclear expression was less frequent in thyroid cancers than that in the normal thyroid gland and hyperplasia. Moreover, the frequency of positive nuclear PATZ1 expression decreased along with the dedifferentiation of thyroid cancer in clinical specimens. Thus, we examined the inhibitory effect of PATZ1 in two DTC cell lines, TPC-1 and FTC-133 *in vitro*.

**Figure 5:**
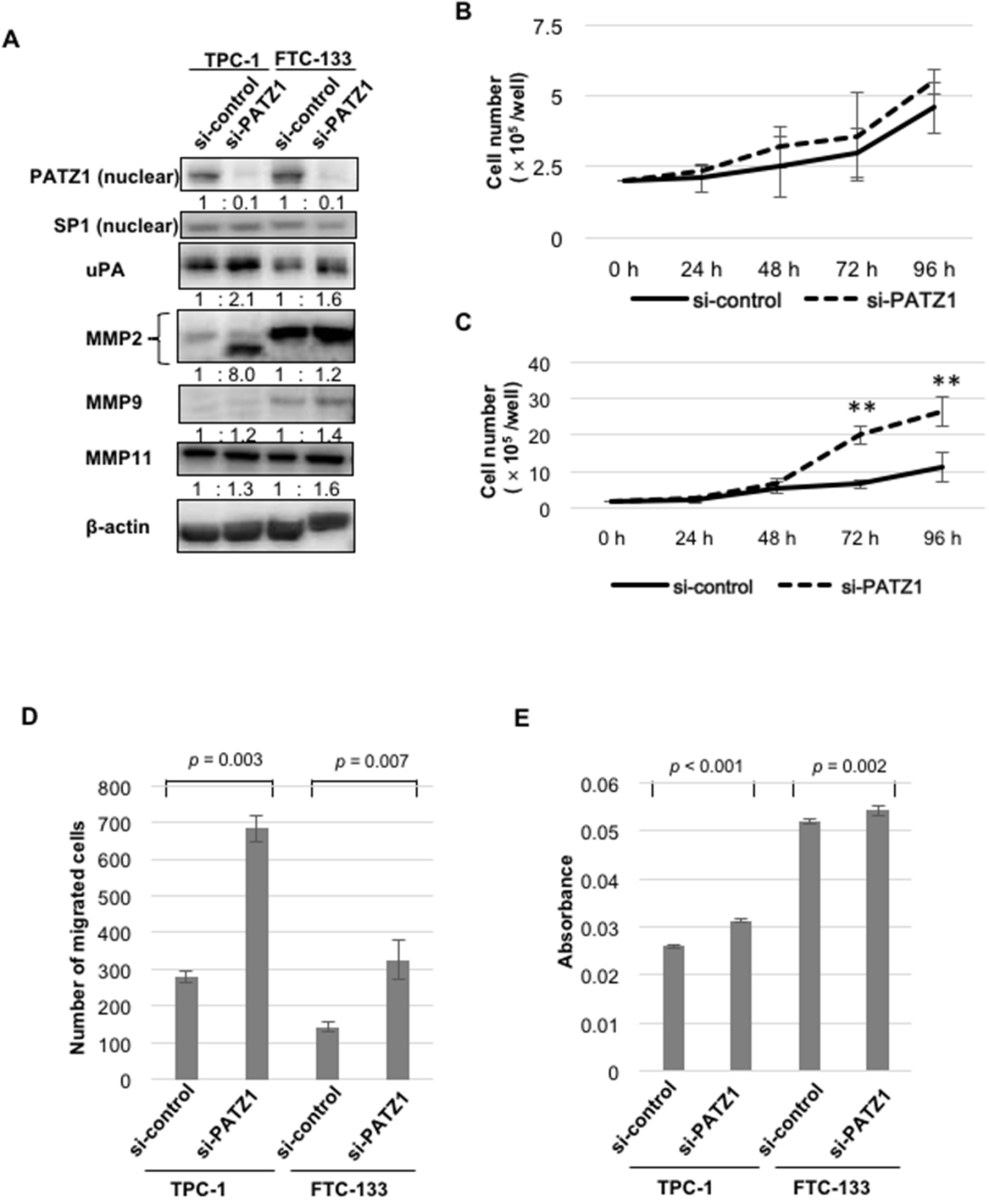
Effects of PATZ1 knockdown on the expression of proteinases, cell proliferation, migration, and invasion in DTC cell lines **(A)** The expression of PATZ1, uPA, and MMPs in DTC cell lines (TPC-1 and FTC-133) transfected with control siRNA (si-control) or siRNA targeting PATZ1 (si-PATZ1). A representative western blot is shown. SP1 and β-actin were used as internal loading control for nuclear extract and whole cell lysate, respectively. **(B)** Cell proliferation assay for TPC-1 cells transfected with si-control or si-PATZ1. The number of cells was counted every 24 h until 96 h after seeding the cells. **(C)** Cell proliferation assay for FTC-133 cells transfected with si-control or si-PATZ1. **p < 0.001. **(D)** Chamber migration assay for DTC cell lines transfected with si-control or si-PATZ1. The number of migrated cells is shown in the bar chart. **(E)** Chamber-invasion assay for DTC cell lines transfected with si-control or si-PATZ1. The cells that invaded through the transwell chamber coated with collagen type IV were counted as described in the Materials and Methods. The absorbance values are shown in the bar chart.

Silencing of nuclear PATZ1 expression by siRNA was confirmed by western blot analysis (Figure 5A). When nuclear expression of PATZ1 was silenced by siRNA, the expression of uPA, MMP2, MMP9, and MMP11 increased in TPC-1 and FTC-133. With regard to cell proliferation, PATZ1 silencing promoted the proliferation of TPC-1 (Figure 5B) and FTC-133 (Figure 5C). With regard to cell motility, PATZ1 knockdown significantly increased cell migration in both cell lines (Figure 5D). Moreover, a significant increase in the invasion ability was observed in both cell lines knocked down for PATZ1 (Figure 5E). These data suggest that PATZ1 knockdown promotes the proliferation of thyroid cancer cells and increases the motility of cancer cells through upregulation of uPA and MMPs.

### Effects of exogenous PATZ1 in anaplastic thyroid cancer cell lines

As nuclear PATZ1 expression decreased along with the progression of thyroid cancer both in clinical specimens and cell lines, we tested whether forced expression of PATZ1 by transfection of a vector encoding human PATZ1 could inhibit the transition of cells to a more malignant phenotype.

**Figure 6:**
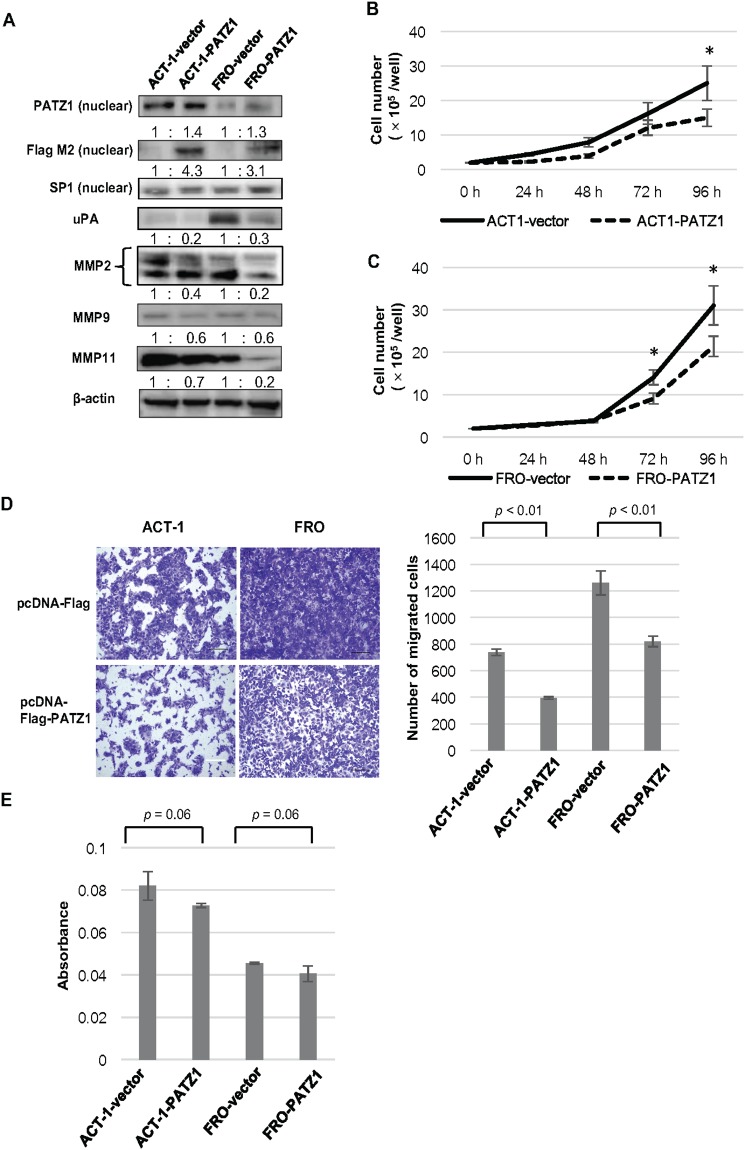
Effects of exogenously introduced PATZ1 in ATC cell lines **(A)** The expression of PATZ1, uPA, and MMPs in ATC cell lines transfected with pcDNA3-FLAG (vector) or pcDNA3-FLAG-PATZ1 (PATZ1). A representative western blot is shown. The nuclear expression of exogenously introduced protein was confirmed by using Flag M2 antibody. SP1 and β-actin were used as internal loading control for nuclear extract and whole cell lysate, respectively. **(B)** Cell proliferation assay for ACT-1 cells transfected with pcDNA3-FLAG or pcDNA3-FLAG-PATZ1. The number of cells was counted every 24 h until 96 h after seeding the cells. *p < 0.05. **(C)** Cell proliferation assay for FRO cells transfected with pcDNA3-FLAG or pcDNA3-FLAG-PATZ1. *p < 0.05. **(D)** Chamber migration assay for ATC cell lines transfected with pcDNA3-FLAG or pcDNA3-FLAG-PATZ1. Representative images of migrating cells stained with crystal violet at 24 h (left panel, scale bar = 200 μm). The number of migrated cells is shown in the bar chart (right panel). **(E)** Chamber-invasion assay for ATC cell lines transfected with pcDNA3-FLAG or pcDNA3-FLAG-PATZ1. The cells that invaded through the transwell chamber coated with collagen type IV were counted as described in the Materials and Methods. The absorbance values are shown in the bar chart.

PATZ1 was exogenously introduced into ACT-1 and FRO cells by transfection of the pcDNA3-FLAG-PATZ1 vector. *PATZ1* mRNA expression levels in the cells transfected with PATZ1 expression vector were increased compared to that in the cells transfected with control vector (data not shown). The nuclear expression of exogenous PATZ1 was confirmed by assessing the expression of the FLAG tag by western blot analysis (Figure 6A). The exogenously introduced PATZ1 decreased the expression of uPA, MMP2, MMP9, and MMP11 both in ACT-1 and FRO cells.

The effect of exogenously introduced PATZ1 on cell proliferation was determined in ATC cell lines. A significant inhibition of cell proliferation was observed in cells transfected with PATZ1 compared to that in cells transfected with control vector in both cell lines (Figure 6B, 6C).

With regard to cell motility, the exogenously introduced PATZ1 significantly decreased the migration of ACT-1 and FRO cells (Figure 6D). Regarding cell invasion, exogenously introduced PATZ1 reduced the invasion ability of ACT-1 and FRO cells (Figure 6E).

These data indicate that forced expression of nuclear PATZ1 inhibits cell proliferation, migration, and invasion of ATC cell lines in which forced expression of PATZ1 downregulated uPA and MMPs.

### Negative correlation between PATZ1 expression and that of uPA/MMPs in clinical specimens

**Figure 7:**
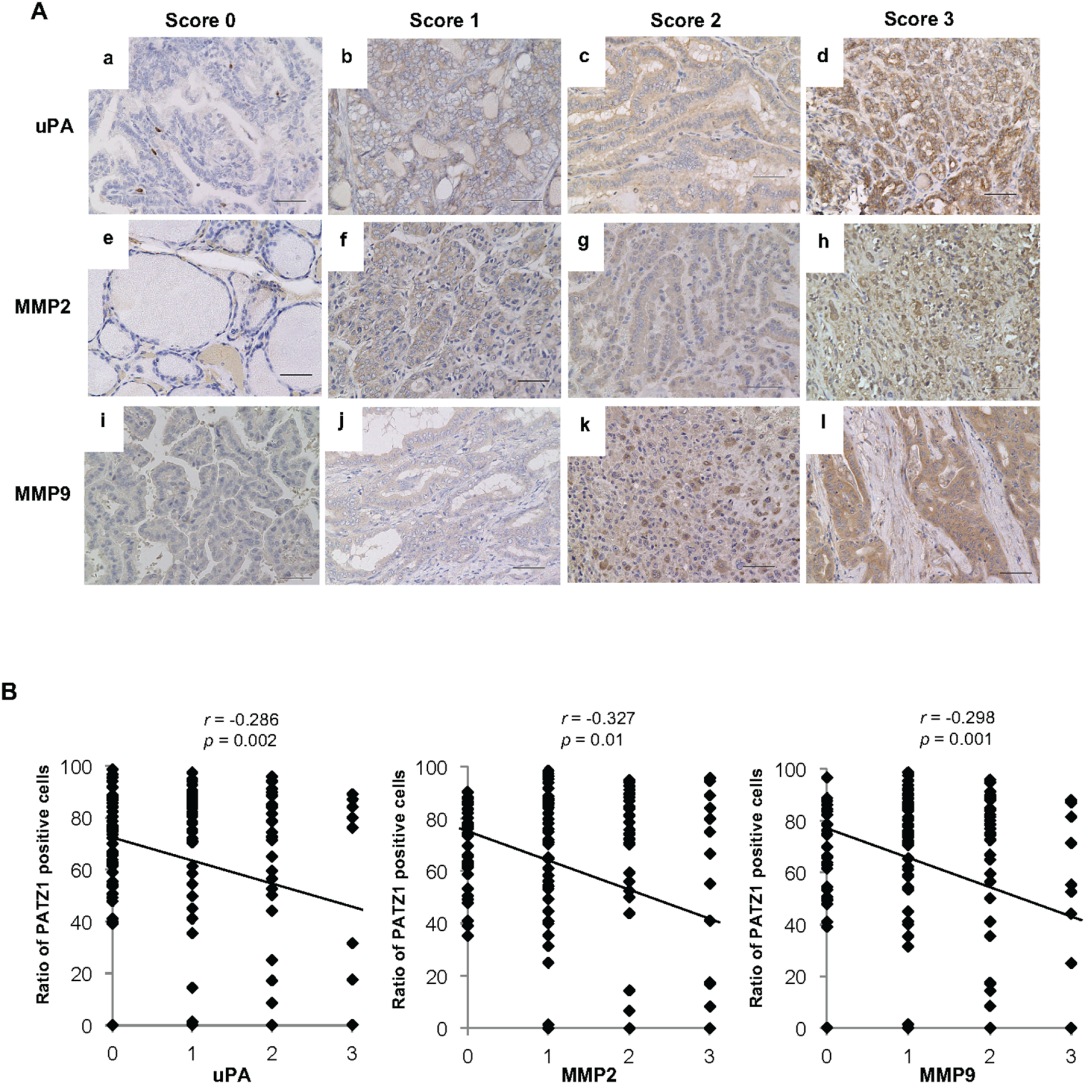
uPA, MMP2, and MMP9 expression in the clinical thyroid tissues **(A)** The expression of uPA, MMP2, and MMP9 in the clinical thyroid tissues was evaluated immunohistochemically on a score of 0 to 3 according to the criteria described in the Materials and Methods. Representative immunohistochemical staining for uPA **(a, b, c,** and **d)**, MMP2 **(e, f, g,** and **h)**, and MMP9 **(i, j, k,** and **l)** is shown. Scale bar = 50 μm. **(B)** Correlation between PATZ1 expression and uPA (a), MMP2 (b) and MMP9 (c) in the clinical thyroid tissues. The expression levels of uPA, MMP2, and MMP9 were classified into four groups, and the ratio of PATZ1 positive cells is plotted in the chart. Correlations between nuclear PATZ1 and uPA or MMPs were analyzed by Spearman’s rank correlation coefficient.

Modification of PATZ1 expression altered the expression of uPA and MMPs in normal thyroid follicular epithelial cells and thyroid cancer cells *in vitro*. Thus, we evaluated the expression of uPA, MMP2, and MMP9 in clinical specimens by immunohistochemistry (Figure 7A, 7B). The expression of uPA, MMP2, and MMP9 was negative in almost all NT and AG, while the ratios of positive uPA, MMP2, and MMP9 expression were high in thyroid cancer specimens (Table 2). Furthermore, a negative correlation was observed between nuclear PATZ1 expression and uPA, MMP2, or MMP9 expression in clinical specimens.

**Table 2:** Expression of PATZ1, uPA, MMP2, and MMP9 in clinical specimens

Histological type		NT (N = 50)	AG (N = 18)	FA (N = 5)	PTC (N = 39)	FTC (N = 8)	PDTC (N = 12)	ATC (N = 28)
**PATZ1**	positive	50 (100%)	18 (100%)	4 (80%)	35 (89.7%)	5 (62.5%)	7 (58.3%)	3 (10.7%)
	negative	0 (0%)	0 (0%)	1 (20%)	4 (10.3%)	3 (37.5%)	5 (41.7%)	25 (89.3%)
**uPA**	positive	0 (0%)	17 (94.4%)	4 (80%)	19 (48.7%)	6 (75%)	12 (100%)	17 (60.7%)
	negative	50 (100%)	1 (5.6%)	1 (20%)	19 (48.7%)	2 (25%)	0 (0%)	5 (17.9%)
**MMP2**	positive	0 (0%)	2 (11.1%)	4 (80%)	29 (74.4%)	6 (75%)	12 (100%)	23 (82.1%)
	negative	50 (100%)	16 (88.9%)	1 (20%)	10 (25.6%)	2 (25%)	0 (0%)	5 (17.9%)
**MMP9**	positive	1 (2%)	2 (11.1%)	1 (20%)	30 (76.9%)	5 (62.5%)	10 (83.3%)	20 (71.4%)
	negative	49 (98%)	16 (88.9%)	4 (80%)	9 (23.1%)	3 (37.5%)	2 (16.7%)	4 (14.3%)

NT; normal thyroid gland, AG; adenomatous goiter, FA; follicular adenoma, PTC; papillary thyroid cancer, FTC; follicular thyroid cancer, PDTC; poorly differentiated thyroid cancer, ATC; anaplastic thyroid cancer.

### Expression of PATZ1 and p53 in thyroid tumor specimens

**Figure 8:**
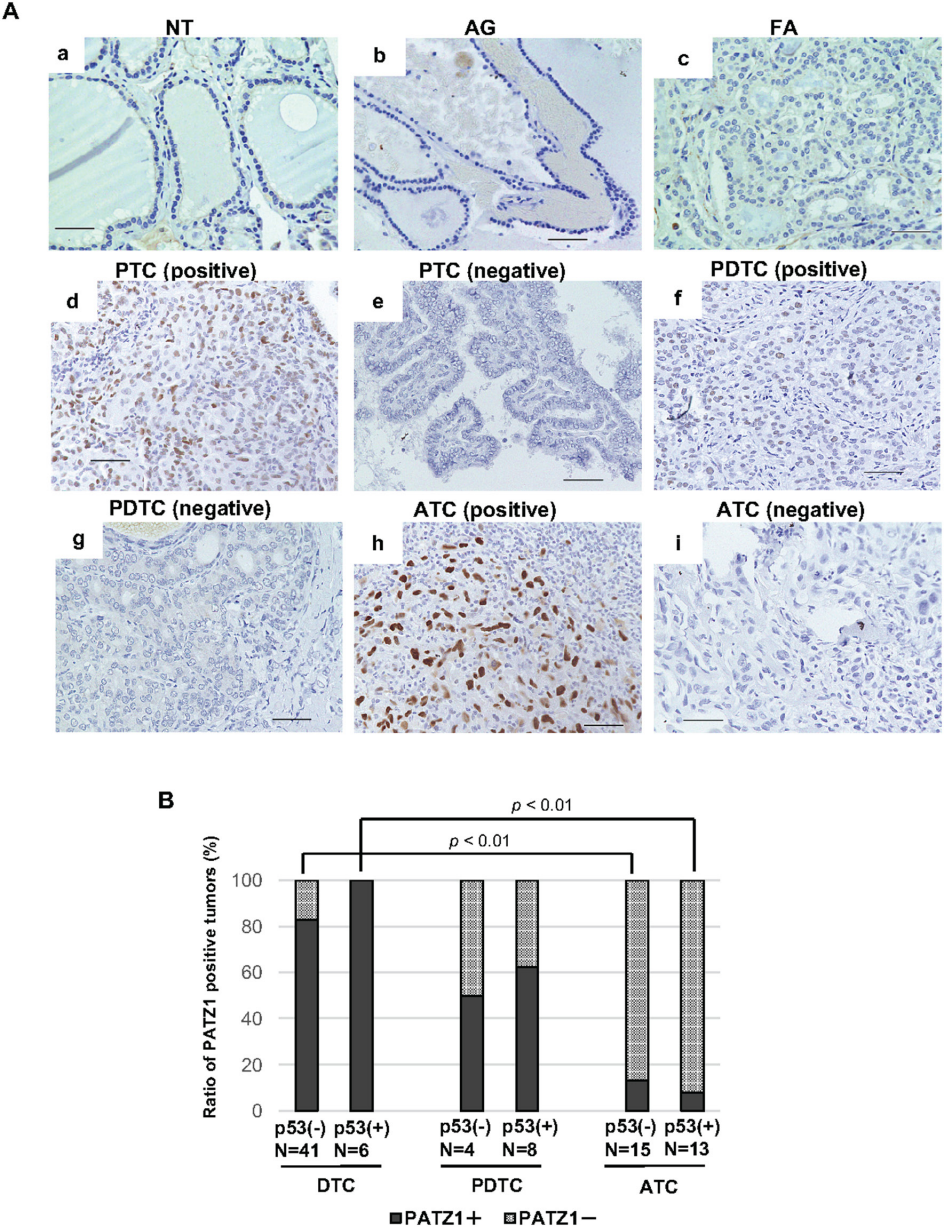
PATZ1 and p53 expression in the clinical thyroid specimens **(A)** p53 expression in the clinical thyroid specimens. Representative images of immunohistochemical analysis of p53 expression are shown; normal thyroid gland (NT) **(a)**, adenomatous goiter (AG) **(b)**, follicular adenoma (FA) **(c)**, papillary thyroid cancer (PTC) (**d**; positive, **e**; negative), poorly differentiated thyroid cancer (PDTC) (**f**; positive, **g**; negative), and anaplastic thyroid cancer (ATC) (**h**; positive, **i**; negative). Scale bar = 50 μm. **(B)** The ratio of PATZ1 positive tumors in differentiated thyroid cancer (DTC), PDTC, and ATC according to p53 status. The percentage of nuclear PATZ1 positive tumors (filled bar) and that of PATZ1 negative tumors (grid bar) are shown in the bar chart.

In the present study, PATZ1 tumor suppressor function was observed regardless of p53 status in thyroid cancer cell lines, we analyzed the expression of PATZ1 and p53 in clinical thyroid tumor tissues. Representative findings of p53 staining are shown in Figure 8A. As shown in Table 3, all NT, AG, and FTC were negative for p53. A positive nuclear staining for p53 was observed in 6 out of 39 PTC (15.4%), in 8 out of 12 PDTC (66.7%), and in 13 out of 28 in ATC (46.4%). Figure 8B demonstrates the ratio of positive nuclear PATZ1 tumors classified according to p53 status in thyroid cancer tissues. Nuclear PATZ1 expression was negative in most DTC tissues, while the ratio of negative nuclear PATZ1 tumors decreased in PDTC and ATC compared with that in DTC regardless of nuclear p53 expression. Additionally, the ratio of nuclear PATZ1 positive tumors was significantly decreased in ATC. Thus, nuclear PATZ1 expression decreased along with the dedifferentiation of thyroid cancer regardless of p53 status in clinical specimens.

**Table 3:** Expression of nuclear p53 in thyroid tumors

Histological type		NT (N = 50)	AG (N = 18)	FA (N = 5)	PTC (N = 39)	FTC (N = 8)	PDTC (N = 12)	ATC (N = 28)
**p53**	positive (%)	0 (0)	0 (0)	2 (40.0)	6 (15.4)	0 (0)	8 (66.7)	13^*#^ (46.4)
	negative (%)	50 (100)	18 (100)	3 (60.0)	33 (84.6)	8 (100)	4 (33.3)	15 (53.6)

NT; normal thyroid gland, AG; adenomatous goiter, FA; follicular adenoma, PTC; papillary thyroid cancer, FTC; follicular thyroid cancer, PDTC; poorly differentiated thyroid cancer, ATC; anaplastic thyroid cancer.

*p < 0.01 *vs*. NT/AG, ^#^*p* < 0.01 *vs*. DTC (PTC/FTC).

## DISCUSSION

The mechanisms underlying carcinogenesis of thyroid epithelial cells have not been fully elucidated. Chiappetta *et al.* demonstrated that PATZ1 expression is negatively associated with thyroid cancer progression in clinical specimens. They also demonstrated that PATZ1 could play a tumor suppressor role in thyroid cancer, mainly involved in the late stage of carcinogenesis by using thyroid cancer cell lines [20]. Consistent with their study, we found that PATZ1 nuclear expression was less frequent with the progression of thyroid cancer, while, as expected, PATZ1 localized in the nucleus of epithelial cells in normal thyroid tissue and hyperplasia. From these findings, we hypothesized that PATZ1 might be involved in the early stage of carcinogenesis of thyroid cancer as well as in the late stage of cancer progression. Hence, we used an immortalized normal thyroid follicular epithelial cell line in addition to thyroid cancer cell lines in the present study. Consequently, we demonstrated for the first time that PATZ1 is involved in, not only dedifferentiation of thyroid cancer cells, but also in the transition of normal thyroid follicular epithelial cells to malignant phenotype, suggesting a tumor suppressor role of PATZ1 in thyroid follicular epithelial cells. Moreover, we demonstrated that PATZ1 negatively regulates the expression of proteinases such as uPA and MMPs that are important for the degradation of the ECM in normal follicular epithelial cells and thyroid cancer cells and that the transition to malignant phenotype induced by loss of nuclear PATZ1 is partly attributed to the promotion of cellular migration and invasion activity by upregulation of these proteinases in thyroid follicular epithelial cells.

Although previous studies reported a cancer-related role for PATZ1, how PATZ1 is involved in the process of carcinogenesis has not been fully elucidated. In addition, both suppressor and promoter functions have been demonstrated for PATZ1 [14, 16, 19]. Pero *et al.* recently reported the development of several tumors, including BCL6-expressing Non-Hodgkin lymphoma, sarcoma, and hepatocellular carcinoma, in PATZ-1-knockout mice and indicated that PATZ1 acts as a tumor suppressor [27]. On the other hand, it has been reported that PATZ1 knockdown by siRNA either inhibits the growth of colorectal carcinoma cells or increases the sensitivity of glioma cell lines to apoptotic stimuli, which indicates the tumor promoter function of PATZ1 [15, 16]. Our present study demonstrated that PATZ1 acts as a tumor suppressor in thyroid follicular epithelial cells and plays an indispensable role in the progression of thyroid cancer, suggesting that the function of PATZ1 might depend on cell types in various organs.

In the present study, we used Nthy-ori 3-1 cells to test the tumor suppressor function of PATZ1. Nthy-ori 3-1 cells are thyroid follicular epithelial cells artificially immortalized by transfection of SV40 large T gene [28]. This is one of few human normal thyroid follicular epithelial cell lines available at present. Nthy-ori 3-1 cells maintain the basic functions of thyroid follicular epithelial cells such as sensitivity to thyroid-stimulating hormone (TSH), iodide-trapping activity, and thyroglobulin production activity, but the cells are non-tumorigenic in nude mice. On the other hand, a previous study reported that Nthy-ori 3-1 cells present migration and invasion ability *in vitro* [29]. Hence, Nthy-ori 3-1 cells are useful for experiments analyzing the regulation of cell growth, thyroid functions, or mechanisms of carcinogenesis. Recently, Kim e*t al.* developed Nthy-ori 3-1 cells expressing either wild-type or mutant *BRAF*V600E using a MCSV promoter-based lentivirus system and reported the results of exploration of biological and genomic alterations caused by *BRAF*V600E mutation in Nthy-ori 3-1 cells [13]. In the present study, we detected PATZ1 in the nucleus of Nthy-ori 3-1 cells, which was consistent with the localization of PATZ1 in normal follicular epithelial cells in clinical tissues, and observed that both morphological alteration and increase of cellular motility was induced by PATZ1 silencing in Nthy-ori 3-1 cells. Consequently, our results obtained in Nthy-ori 3-1 cells may indicate the involvement of PATZ1 as a tumor suppressor in the early stage of carcinogenesis in thyroid follicular epithelial cells.

Recent studies demonstrated a functional interaction between PATZ1 and p53 [13, 18] Valentino *et al.* demonstrated that PATZ1 interacts with p53 and regulates the transcription of p53-regulated genes [13] and PATZ1 function depends on p53 status; i.e., PATZ1 enhances the expression of the genes regulated by p53 and functions as a tumor suppressor in the presence of wild-type p53. However, the absence of p53 leads PATZ1 to inhibit the same genes, playing an oncogenic role. Thus, they proposed an oncogenic or anti-oncogenic role for PATZ1 in carcinogenesis depending on the cellular context. In the present study, PATZ1 silencing induced oncogenic effects in Nthy-ori 3-1 cells. As Nthy-ori 3-1 cells express wild-type p53, the oncogenic effects observed by PATZ1 silencing was consistent with the tumor suppressor function in the presence of wild-type p53 proposed by Valentino *et al.*

On the other hand, oncogenic effects such as upregulation of cell proliferation, migration, and invasion induced by PATZ1 silencing in the present study were more significant in DTC cell lines (TPC-1 and FTC-133) than those observed in ATC cell lines (ATC-1 and FRO) regardless of p53 status. Furthermore, tumor suppressor effects such as inhibition of proliferation and cellular motility induced by exogenously introduced PATZ1 were more clearly observed in ATC cell lines compared with those observed in DTC cell lines irrespective of the p53 status. Chiappetta *et al.* demonstrated that restoration of PATZ1 expression in thyroid cancer cell lines derived from dedifferentiated cancers significantly inhibited their malignant behaviors, including *in vitro* proliferation, anchorage-independent growth, migration, and invasion as well as *in vivo* tumor growth [20]. Our data together with Chiappetta *et al.* observation suggest that PATZ1 functions as a tumor suppressor in thyroid follicular epithelial cells as well as in thyroid cancer originating from follicular epithelial cells, and its oncogenic function does not depend on the p53 status. In the present study, PATZ1 nuclear expression was lower in ATC cell lines compared to that in TPC-1 cells. In addition, the inhibition of malignant behaviors by exogenously introduced PATZ1 was more significant in ATC cell lines than those in DTC cell lines both in our study and in Chiappetta *et al.*’s study. In contrast, the promotion of malignant behaviors induced by PATZ1 silencing was most drastic in Nthy-ori 3-1 cells than in DTC cell lines. These data suggest that the malignant behaviors induced by PATZ1 silencing may depend on the expression level and localization of endogenous PATZ1 in each cell.

In clinical specimens, most p53 positive DTC presented with positive nuclear staining for PATZ1. Although the frequency of p53 positive tumors increased in PDTC and ATC, the frequency of positive nuclear PATZ1 tumors decreased with the progression of dedifferentiation regardless of p53 status. These findings together with the results obtained *in vitro* also suggest that an interaction between PATZ1 and p53 may not be necessary for PATZ1 tumor suppressor function.

One of the most vital aspects of cancer cells is their enhanced ability to migrate and invade into adjacent tissues. These events include significant changes in cell morphology as well as degradation of the ECM. It is well-known that urokinase-type plasminogen activating system and members of the MMP family play indispensable roles in the ECM proteolysis as well as in cell migration and adhesion. With regard to the thyroid, Ulisse *et al.* reported that malignant transformation of human thyroid follicular epithelial cells is associated with the augmented expression of uPA both at the mRNA and protein levels in clinical specimens [30]. In addition, several studies documented an increased expression and/or activity of members of the MMP family in thyroid cancer [31]. Among the MMP family members, an increased expression and activity of both MMP2 and MMP9, which use type IV collagen as their substrate, has been reported to correlate with enhanced invasion of thyroid cancer cells [32–34]. Furthermore, MMP11 expression, which is also named stromelysin-3 and is correlated with cell migration, tissue morphogenesis, and apoptosis during the development and invasion of tumor cells [35–37], is linked to aggressive characteristics of PTC [38, 39]. In the present study, a drastic upregulation of the expression and activity of uPA and MMP2, 9, and 11 was induced by PATZ1 silencing in the immortalized normal follicular epithelial cell line, Nthy-ori 3-1 cells. In addition, PATZ1 negatively regulated the expression of uPA and these MMPs in thyroid cancer cell lines. Moreover, immunohistochemical analysis of the clinical specimens supported the data obtained *in vitro*.

A potential tumor suppressor role of PATZ1 has been demonstrated in previous studies. Our present study provides clear evidence that PATZ1 is involved in the regulation of the expression of uPA and MMPs during the process of carcinogenesis and progression of thyroid cancer. Although we are aware that spontaneous *in vivo* thyroid cancer models are appropriate to elucidate the involvement of PATZ1 in the early stage of carcinogenesis in thyroid follicular epithelial cells and further studies are required to elucidate the precise mechanisms of regulation of uPA and MMPs by PATZ1 in thyroid follicular epithelial cells, our study is the first report demonstrating that PATZ1 exerts its tumor suppressor role by regulating proteolytic enzymes important for fundamental features of tumor cells such as migration and invasion.

In conclusion, we demonstrated that PATZ1 plays an important role in both the process of carcinogenesis of thyroid follicular epithelial cells and in the progression and dedifferentiation of thyroid cancer. In addition, our data indicate that the increase in the proteolytic activities of uPA and MMPs is one of the pro-oncogenic mechanisms associated with PATZ1 depletion. Further analyses of PATZ1 and related molecules may lead to the development of novel therapeutic strategies to prevent the progression of thyroid cancer.

## MATERIALS AND METHODS

### Clinical specimens

This study was conducted according to the ethical guidelines of the Declaration of Helsinki, and specific approval was obtained from the Ethics Committee of Shinshu University School of Medicine (approval number #507). The patients provided written informed consent for the use of their specimens for the study, and the Ethics Committee approved this consent procedure. The studied specimens were obtained from 82 patients with thyroid cancers who were diagnosed and treated in Shinshu University Hospital from 1996 to 2014.

### Cell lines

Immortalized human thyroid epithelial cell line, Nthy-ori 3-1, was purchased from DS Pharma Biomedical (Osaka, Japan) [28]. Two DTC cell lines (TPC-1 and FTC-133) and two ATC cell lines (FRO and ACT-1) were used in the study. TPC-1, which originated from PTC, and FRO cells were a gift from Dr. Yamashita at Nagasaki University [40]. FTC-133, which originated from FTC, was kindly provided by Dr. Takeda at the Fourth Department of Internal Medicine, Shinshu University School of Medicine [41]. ACT-1 was established by Dr. Ohara at Tokushima University and was a kind gift [23]. All cell lines were maintained in RPMI 1640 (Sigma-Aldrich, Saint Louis, MO, USA) supplemented with 10% heat-inactivated fetal bovine serum (FBS) at 37°C under 5% CO_2_. Cell number was counted by using Cytorecon (GE healthcare, Tokyo, Japan).

### Immunofluorescence analysis

The cells were grown on cover glasses and fixed in 4% paraformaldehyde/PBS for 30 min and 0.1% Triton X-100 for 30 min. After treatment with 1% bovine serum albumin (BSA), they were exposed to primary antibodies against PATZ1 (#ab154025, Abcam, Cambridge, UK) or p53 (#sc-6243, Santa Cruz Biotechnology, Dallas, TX, USA). Subsequently, the cells were treated with Alexa Fluor 488 goat anti-rabbit IgG (Abcam) for PATZ1 or Alexa Fluor 594 goat anti-rabbit IgG (Abcam) for p53 and Prolong Gold Antifade Reagent with DAPI (Life technologies, Carlsbad, CA, USA). Fluorescence was observed by using a fluorescence microscope (Keyence, Osaka, Japan).

### Immunohistochemical staining and evaluation

Formalin-fixed, paraffin-embedded (FFPE) 3-μm-thick sections were obtained from clinical specimens. Sections were deparaffinized in xylene, rehydrated, and treated with peroxide in 100% methanol for 30 min to inhibit endogenous peroxidase activity. For immunohistochemical analysis, slides were heated for antigen retrieval in 10 mM of sodium citrate (pH 6.0). Sections were subsequently exposed to specific antibodies for PATZ1 (#ab154025, Abcam), p53 (#sc-6243, Santa Cruz Biotechnology), uPA (#ab169754, Abcam), matrix MMP2 (#sc-10736, Santa Cruz Biotechnology), and MMP9 (Fuji chemical industries Ltd., Toyama, Japan), or isotype-matched controls at appropriate dilutions. Then, sections were incubated with Histofine Simple Stain MAX PO (MULTI) (Nichirei Biosciences, Tokyo, Japan). The staining was revealed by using diaminobenzidine (Nichirei Biosciences), and sections were counterstained with aqueous hematoxylin. The number of cells showing PATZ1 positive or p53 positive nuclear staining in each high-power field (HPF) (×400) was quantitated, and more than 30% staining was considered positive for both PATZ1 and p53. The expression of uPA, MMP2, and MMP9 was evaluated based on cytoplasmic expression by using immunostaining scores (none = 0, weak = 1, moderate = 2, strong = 3). Scores 0 and 1 were considered negative and scores 2 and 3 were considered positive. As a positive control for PATZ1, one normal thyroid gland was used in each immunohistochemical staining assay., One ATC specimen apparently negative for both nucleic and cytoplasmic staining was used as a negative control for PATZ1. As a positive control for p53, we used one ATC specimen apparently positive for nuclear staining in each immunohistochemical staining assay. One normal thyroid gland was used as a negative control. As controls for the antibodies, we used isotype specific non-immune immunoglobulins at the same concentration as the primary antibody.

### Transfection of small interfering RNA (siRNA)

siRNA for PATZ1 (M-013539-00-0005) and the control siRNA (D-001206-14-05) were purchased from GE healthcare life sciences (Pittsburgh, PA, USA). Transfection of each siRNA (20 nM) was performed by using Lipofectamine RNAi-MAX (Invitrogen, Carlsbad, CA, USA). After 48 h of transfection, the medium was replaced and the cells were further incubated for 24 h. After incubation, the cells were counted, cell lysates were obtained, and morphological changes, migration ability, and invasion ability were assessed.

### Plasmid DNAs

PATZ1 (variant 2, PATZ1 long A isoform) cDNA was amplified from the cDNA library from heart tissue using the following primers: 5′-GAATTCATGGAGCGGGTGAACGACGCTTC-3′ and 5′-TGTACATCATTTCCCTTCAGGCCCCATGGG-3′. After confirming the sequence (CCDS13895), the PATZ1 cDNA was subcloned into the *Eco*RI site of pcDNA3-FLAG, and the expression vector was designated as pcDNA3-FLAG-PATZ1. The pcDNA3-FLAG was used as a control. pcDNA3-FLAG-PATZ1 or pcDNA3-FLAG was transfected into each cell line using FuGENE HD Transfection Reagent (Roche Diagnostics, Basel, Switzerland) following the manufacturer’s protocol.

### Total RNA extraction and quantitative real-time RT-PCR

Total RNA was extracted by using an RNeasy Mini kit (Qiagen, Alameda, CA, USA) according to the manufacturer’s instructions. Taqman Gene Expression Assays for PATZ1 (# Hs00204880_ml), β-actin (Hs99999903_ml) were purchased from Applied Biosystems (Carlsbad, CA, USA) and mRNA levels were qualified in triplicate using Applied Biosystems 7300 Real-Time PCR system (Applied Biosystems).

### Western blot analysis

Proteins were isolated from the cells and western blot analyses were performed as previously described [42]. Ten micrograms of protein were loaded and membranes were probed with antibodies against PATZ1 (#ab154025, Abcam), uPA (#ab169754, Abcam), MMP2 (#sc-10736, Santa Cruz Biotechnology), MMP9 (Fuji chemical industries Ltd.), MMP11 (#ab52904, Abcam), Flag M2 (#F-3165, Sigma-Aldrich), and p53 (#sc-6243, Santa Cruz Biotechnology) and visualized by the enhanced chemiluminescence protocol (Amersham Biosciences, Piscataway, NJ, USA). We used β-actin (#A5441, Sigma-Aldrich) for whole lysate and SP1 (#ab13405-50, Abcam) for nuclear lysate as loading controls. Each experiment was independently repeated at least three times, and one representative blot is presented in the figures. The quantitative analysis was performed by using the ChemiDoc XRS and Quantity One software (Bio-Rad Laboratories, Tokyo, Japan).

### Cell proliferation assay

Cells (2 × 10^5^ cells/well) were seeded on a 6-well plate with 500 μL of RPMI 1640 containing 10% FBS. The number of cells was counted by using Cytorecon (GE healthcare, Tokyo, Japan) from 24 h until 96 h after seeding the cells.

### Scratch wound assay

Cells were seeded on a 6-well plate and allowed to reach confluence. After scratching the bottom of the well with a pipette tip, the cell monolayer was washed with PBS to remove detached cells. The distance of the scratch wound was evaluated at 6 h and 16 h after the scratch wound was performed. We measured the length of the gap as 100 points for each sample and calculated an average [43]. Each experiment was performed in triplicate.

### Chamber migration assay

Falcon cell-culture insert (Fisher scientific, Pittsburgh, PA, USA) with an 8-μm pore membrane was placed in six well plates. Cells (2 × 10^5^ cells/insert) were seeded onto the upper chamber without FBS, and 500 μL of RPMI 1640 containing 10% FBS was loaded onto the bottom chamber. After 24 h of incubation, the non-migrating cells were removed from the upper surface of the membrane by gently scrubbing by using cotton tipped swab. Cells on the lower surface of the membrane were fixed with methanol and then stained with 0.2% crystal violet followed by two washes with distilled water. Inserts were air-dried. Thereafter, we counted the number of cells under a microscope at 100× magnification in five randomly selected fields for each sample, and each experiment was repeated at least three times.

### Chamber invasion assay

We used Cultrex 24-well collagen IV cell invasion assay kit (Trevigen, Gaithersburg, MD, USA). Transwell cell culture inserts with an 8-μm pore membrane were coated with collagen type IV. Cells (2 × 10^5^ cells/insert) were seeded onto the upper chamber without FBS, and 500 μL of RPMI 1640 containing 10% FBS was loaded onto the bottom chamber. After 48 h of incubation, the cell dissociation solution was added onto the bottom chamber and incubated 1 h. The cell dissociation solution was quantitated with a microplate reader (Wako, Osaka, Japan) and the SoftMax Pro 5.1 software (Wako, Osaka, Japan). Each experiment was performed at least three times.

### Morphological change on matrigel

Two hundred microliters of chilled Matrigel (Corning, NY, US) were used to coat each well of a 12-well plate and allowed to polymerize at 37°C for 1 h. Cells (7.5 × 10^4^ cells/well) were seeded on the Matrigel in 1 mL of RPMI 1640 containing 10% FBS. After 48 h of incubation, the morphology of the cells was analyzed.

### F-actin detection

Nthy-ori 3-1 cells were cultured on coverslips for 24 h. The cells were treated with PBS containing 4% PFA, permeabilized with 0.1% Triton X-100 in PBS, and supplemented with 100 nmol/L rhodamine-phalloidin (Cytoskeleton, Inc., Denver, CO, USA) in PBS containing 0.1% BSA for 1 h at room temperature. Lastly, the cells were mounted with Prolong Gold Antifade Reagent with DAPI (Life technologies). Signals were observed under a fluorescence microscope (Keyence).

### Fibrin zymography

uPA activity was examined by fibrin zymography. Samples and urokinase were subjected to electrophoresis on a 10% polyacrylamide gel containing bovine fibrinogen (0.55 mg/mL) (Sigma-Aldrich) and thrombin (0.056 NIH U/mL) (Sigma-Aldrich). The gels were washed and incubated for 48 h in an incubation buffer containing 0.5 M glycine-HCl (pH 8.4) at 37°C. After staining the gels with Coomassie blue, the gels were scanned using the ChemiDoc XRS (Bio-Rad Laboratories). The experiment was performed at least three times.

### Gelatin zymography

MMP2 and MMP9 activities were measured with Gelatin Zymo-Electrophoresis kit (Primary Cell, Sapporo, Japan) according to the manufacturer’s protocol. In brief, 10 μL of each sample were loaded for electrophoresis. The gels were washed and incubated for 48 h in the incubation buffer at 37°C. After staining the gels with Coomassie blue, the gels were scanned using the ChemiDoc XRS (Bio-Rad Laboratories). Each experiment was performed at least three times.

### Statistical analysis

Statistical analyses were performed by using SPSS Statistics version 20.0 (IBM Japan, Tokyo, Japan). The chi-square test was used for immunohistochemical analysis of the clinical specimens. Data from the real time RT-PCR, proliferation assay, scratch assay, migration assay, and invasion assay were examined by Student *t*-test with a *P*-value of less than 0.05 considered statistically significant. Correlation between nuclear PATZ1 and uPA or MMPs in the clinical specimens was analyzed by Spearman’s rank correlation coefficient.
